# Oculomotor markers of functional impairment in traumatic brain injury using simple and predictive visual tracking

**DOI:** 10.3389/fneur.2026.1822886

**Published:** 2026-07-10

**Authors:** Shimrit Shani, Oren Kadosh, Yaron Sacher, Keren Cismariu-Potash, Yoram S. Bonneh

**Affiliations:** 1 School of Optometry and Vision Science, Bar-Ilan University, Ramat Gan, Israel; 2 The Leslie and Susan Gonda, Multidisciplinary Brain Research Center, Bar-Ilan University, Ramat Gan, Israel; 3 Department of Traumatic Brain Injury, Loewenstein Rehabilitation Medical Center, Ra’anana, Israel; 4 Sackler Faculty of Medicine, Tel Aviv University, Ramat Aviv, Israel

**Keywords:** closed-loop, eye-tracker, FIM (Functional Independence Measure), prediction, smooth pursuit, TBI - traumatic brain injury

## Abstract

**Background:**

Oculomotor function is a sensitive marker of neurological impairment, and smooth pursuit deficits have been reported across multiple neurological disorders. However, the oculomotor deficits following traumatic brain injury (TBI) remain incompletely characterized, particularly in relation to current functional status during rehabilitation.

**Methods:**

In this study, we employed a bedside, head-free eye-tracking paradigm based on short, repeated linear tracking segments, to assess oculomotor dysfunction in 30 TBI patients undergoing inpatient rehabilitation and 30 age-matched controls. This design enabled the extraction of multiple complementary oculomotor indices, including saccadic pursuit (catch-up saccade contribution), tracking deviation under occlusion, initial tracking speed, initial catch-up saccade latency, pupil response, and vergence instability.

**Results:**

TBI patients exhibited widespread deficits across these indices compared with controls (AUC = 0.71-0.84). These measures also correlated significantly with functional status as indexed by the Functional Independence Measure (R = 0.39-0.77, p < 0.001) but, importantly, showed no association with initial injury severity as indexed by the Glasgow Coma Scale.

**Conclusion:**

These findings indicate that TBI disrupts multiple components of the oculomotor system, extending beyond basic pursuit gain to predictive tracking, pupil-linked arousal, and binocular coordination. The dissociation between functional status and initial acute injury severity highlights the sensitivity of oculomotor measures to current brain function during rehabilitation. Together, these results support the use of brief, clinic-friendly eye-tracking assessments as a quantitative approach for evaluating current functional status in TBI, with potential future applications for longitudinal monitoring pending further validation.

## Introduction

Traumatic Brain Injury (TBI) is one of the leading causes of death and disability worldwide; unfortunately, its incidence and associated healthcare burden are continuing to rise (1). TBI often results from motor vehicle accidents, falls, or sports injuries, and it encompasses a wide spectrum of outcomes, ranging from mild cognitive disturbances to severe, life-altering impairments. Among the many deficits associated with TBI, vision and oculomotor impairments stand out as both common and disabling, with significant consequences for daily functioning (2, 3). These deficits include disruptions in smooth pursuit eye movements, saccadic accuracy, and visual tracking during occlusions, functions that rely on the intricate coordination of distributed brain networks (4–6). Smooth pursuit eye movements allow the eyes to maintain stable fixation on moving objects by minimizing position errors. These movements involve two mechanisms: an “open-loop” phase, in which the initial movement is generated without feedback, and a “closed-loop” phase, which adjusts the movement based on target feedback. TBI can disrupt both phases, impairing the tracking precision and increasing reliance on compensatory catch-up saccades (7, 8). The pursuit system’s sensitivity to neurological disruptions makes it a valuable tool for assessing the functional impact of TBI. Predictive mechanisms also play a crucial role in smooth pursuit, particularly when tracking objects that become temporarily occluded. More generally, temporal expectation is accompanied by systematic changes in oculomotor behavior (9, 10). Previous research has shown that pursuit eye movements enhance motion prediction, consequently improving accuracy in anticipating target trajectories even when vision is disrupted (11). However, such predictive tracking can be impaired in clinical populations, including TBI patients, where deficits in motion extrapolation may contribute to tracking errors under occlusion. Such impairment was found in chronic mild TBI patients (12).

Several studies have examined visual tracking performance in TBI populations. For example, circular and vertical pursuit tasks have revealed slower tracking speeds, reduced pursuit gain, and more catch-up saccades in TBI patients than in controls (13, 14). Although these studies have demonstrated the utility of eye tracking for detecting group-level differences, they often lack correlations with individual-level clinical severity and provide limited insight into the nuanced impairments associated with mild TBI. Furthermore, although horizontal and vertical saccadic tasks have shown promise in distinguishing TBI patients from controls (15), their applicability for mild cases and their relationship to current functional status during rehabilitation remain underdeveloped.

In this study, we addressed these gaps by conducting a detailed analysis of visual tracking in TBI patients, focusing on smooth pursuit and tracking under occlusion. Rather than the continuous circular or sinusoidal paradigms used in previous work, we employed repeated short linear segments extending from central fixation. This enabled the extraction of six complementary, clinically oriented oculomotor measures: saccadic pursuit (defined here as the cumulative catch-up saccades occurring during smooth tracking), tracking deviation under occlusion, initial tracking speed, initial catch-up latency, initial pupil response, and vergence stability. These measures were designed to capture robust, clinically meaningful markers from a practical, head-free bedside system, remaining compatible with the temporal resolution of the setup. Although the study includes repeated data collection throughout the rehabilitation period, the design remains observational; therefore, associations between oculomotor measures and functional outcomes should be interpreted as correlational rather than causal. Our findings highlight the potential of a brief, accessible eye-tracking approach to quantify oculomotor abnormalities after TBI, while applications to prognosis and recovery monitoring remain preliminary. An earlier version of this work, including additional exploratory analyses, was previously posted as a preprint (16).

## Methods

### Participants

The basic demographic and clinical information for the participants is presented in Table 1. Initially, 33 patients with TBI were recruited for the study. Three participants were excluded due to early technical errors during data acquisition where eye-tracking data were not recorded properly, yielding a final analyzed sample of 30 patients (21 severe, 2 moderate, and 7 mild, based on the Glasgow Coma Scale [GCS]). The Glasgow Coma Scale (GCS) scores reported in this study refer to the initial assessment performed in the acute phase, typically at the scene of injury or upon admission to acute care. Importantly, these scores do not reflect the patients’ level of consciousness or cognitive status at the time of testing, which took place during the rehabilitation phase. At the time of participation, all patients were medically stable, able to provide informed consent, and capable of following task instructions. The subjects were recruited from the Loewenstein Rehabilitation Hospital in Ra′anana, Israel. This study was conducted at the Loewenstein Rehabilitation Medical Center and was approved by the Helsinki Committee of the Loewenstein Hospital. The participants were aged 18–70 (M = 39.7, SD = 17.87), had a minimum corrected visual acuity of 6/12, and were free of existing psychiatric or neurological conditions, bilateral eye or oculomotor problems, involuntary movements of the head and body that interfere with positioning in front of a screen and eye tracking, as well as paralysis of extraocular muscles. The control group, which also included 30 people, was age-matched with the TBI group (M = 40.2, SD = 15.78) and shared the same inclusion criteria. The analyzed TBI cohort included 27 men and 3 women. All participants were proficient in Hebrew and completed the study procedures across separate testing days. All TBI patients were hospitalized in the rehabilitation department for the entire study period and were evaluated by the medical staff to ensure that they could follow the instructions. Three TBI participants were excluded from eye movement analysis due to erroneous recordings. Injury details, GCS scores, and brain imaging findings for the TBI group were obtained from medical records. GCS scores ranged from 3 to 15 (see Table 1 for detailed injury profiles). Clinical and demographic characteristics of the TBI patient group are summarized in Table 2. FIM scores were recorded at rehabilitation admission and at study entrance, defined as the time point at which participants were enrolled in the study during inpatient rehabilitation. The small number of female participants reflects the male predominance of TBI cases reported in prior epidemiological studies (17). All participants provided informed consent and were informed that they would not receive compensation for their participation. All experiments were conducted in accordance with the Helsinki guidelines. Medication status was recorded for antiepileptic treatment (levetiracetam, Keppra). Other medications were prescribed according to standard clinical care and were not experimentally controlled. Patients were medically stable and not under acute sedation at the time of testing.

**Table 1:** Demographic data by age and gender.

Group	*N*	Age (avg ± SD)	Females/Males	FIM_enter_ avg	FIM_leave_ avg	GCS
Control	30	40.2 (±15.5)	15/15	N/A	N/A	N/A
TBI all	33	39.7 (±17.87)	3/30	56.9	101.9	6.87 (±4.75)
Mild	7	46 (±15.49)	2/5	58.8	102 (±18.97)	14.6 (±0.78)
Moderate	3	70	0/3	43.5	77 (±38.2)	11 (1.4)
Severe	23	36.1 (±16.37)	1/22	60.65	104.34 (±20.9)	4.17 (±1.88)

N = number, SD = Standard deviation, TBI = Traumatic Brain Injury, FIM_enter_/FIM_leave_ = Functional Independence Measure (clinical assessment at the beginning (FIM_enter_) and around the end of (FIM_leave_) the hospitalization). The severity level (Mild, Moderate, and Severe) was determined by the Glasgow Coma Scale (GCS).

**Table 2:** Clinical characteristics of the TBI patient group.

Patient no.	Age	Time from injury to rehab admission (d)	GCS (1–15)	FIM admission (18–126)	FIM study entrance	Imaging finding
1	38	49	6	87	87	Bilat P, RT F
2	49	35	7	36	78	Rt PO Contusions, RT SDH and SAH, DAI
3	49	49	3	36	36	Bilat SAH, DAI
4	25	21	15	56	69	Subdural hemorrhage R + L
5	31	73	3	31	76	Rt ICH, Craniectomy
6	36	36	15	68	96	Rt hemispheric SDH, SDH along the falx and posterior tentorium, SAH Lt frontal lobe, and basal cisterns, Rt parietotemporal fracture, Rt temporal bone fractures, Lt fronto-parieto-temporal linear fracture
7	61	19	14	69	74	Bilateral bleeding F + T lobes
8	36	7	15	74	74	Lt SM Cortex
9	38	27	3	69	85	Lt SAH, Lt F EDH, Lt P Contusion
10	70	51	10	20	39	Lt SDH, Craniotomy, Hydrocephalus, and VP shunt insertion
11	67	58	8	38	57	Rt F SDH, Bilat FP SAH
12	60	79	3	41	46	DAI
13	27	32	3	93	93	DAI
14	52	55	15	37	55	Rt SDH
15	70	29	12	67	85	Rt TP Contusion
16	27	24	3	72	90	R Fronto-parieto-occipital
17	24	33	3	63	114	DAI
18	21	73	3	64	64	Rt parietal EDH, Lacunar infarct in the right basal ganglia
19	19	30	3	43	43	Rt F contusion, DAI
20	25	15	8	80	113	Rt SDH, Rt F SAH, Rt F Contusion
21	47	108	4	64	64	Bilat SAH, Lt BG Hemo, Rt Th Hemo
22	18	41	-	46	54	Rt SDH with midline shift
23	45	40	5	82	82	Bilat T EDG, Lt T Contusion
24	69	22	13	40	40	Rt F Contusion
25	25	31	3	74	115	Lt occipital SDH, Cerebral contusions, SAH
26	19	114	3	28	28	Lt F EDH, DAI
27	40	62	3	89	89	Bilateral F, RT Craniectomy
28	43	6	15	68	68	DAI
29	70	19	5	33	41	Bilat F T, DAI
30	27	46	3		74	Lt frontoparietal SDH + SAH

Three recruited participants were excluded from eye-tracking analyses because of poor quality or erroneous data. GCS values represent initial injury severity assessed in the acute phase and do not reflect the patients’ functional or cognitive status at the time of rehabilitation testing. FIM scores were recorded at rehabilitation admission and at study entrance, defined as the time point at which participants were enrolled in the study during inpatient rehabilitation. Time from injury to rehabilitation admission is reported in days.

### Apparatus

The participants were seated in a stationary chair (without wheels) with their heads free in front of a computer display and were tested in their hospital room under normal illumination. The stimuli were presented on a 14” LCD laptop monitor running in Full HD resolution (1920 × 1,080) with a 60 Hz refresh rate, positioned on a standard table. Eye movements and pupil size were recorded using a remote video-based eye-tracking system (Tobii 4C, 90 Hz sampling rate) from a viewing distance of approximately 60 cm. Tobii-based systems operating at comparable sampling rates have previously been used to extract clinically relevant oculomotor biomarkers in neurodegenerative and ophthalmologic populations, including measures of fixation stability, gaze behavior, vergence, saccade rate, and pupil dynamics (18–20). While this 90 Hz rate is well-suited for assessing spatial dispersion and low-frequency tracking behavior, temporally sensitive variables like initial catch-up latency were interpreted cautiously. Specifically, these were treated as trial-averaged timing estimates—which are coarse at the single-trial level but become robust at the subject level through repeated measurements. The stimuli were presented binocularly using an in-house-developed platform for psychophysical and eye-tracking experiments (PSY) developed by Yoram S. Bonneh, running on a Windows PC, which was used in many of our previous studies [e.g., (21–23)].

### Stimuli and procedure

#### Stimuli

##### Experiment 1: smooth pursuit

**Figure 1:**
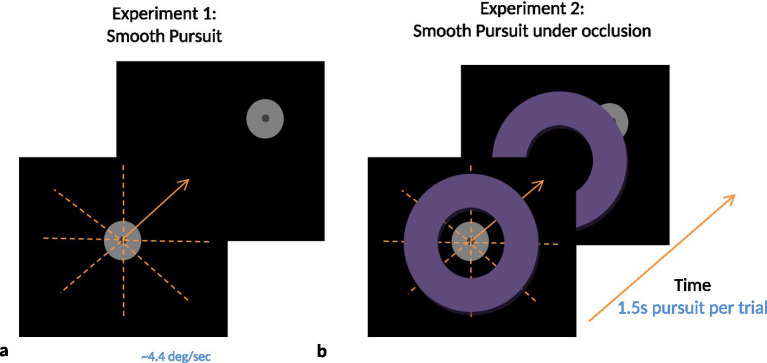
Stimuli were used for the smooth pursuit experiments. **(a)** Simple pursuit. A central fixation cross was shown for 500 ms; then a bright disk appeared and started moving smoothly in one of 8 directions in random order at ~4.4 deg./s speed (horizontal and vertical components are viewed from 60 cm), for 1.5 s per trial, repeated 16 times per run. **(b)** Occluded pursuit. The same sequence as in panel **(a)**; it was shown with the addition of an occluding ring with 3 width sizes (the medium width is shown). A third experiment was identical to the occluded pursuit experiment, except that the ring was black, i.e., invisible.

The stimulus sequence is depicted in Figure 1a. A fixation cross (~1 dva) was presented on a black background for 0.5 s. A white target disc (~1.5 dva diameter, 35.6 cd/m^2^ in luminance) appeared at fixation and moved from the center outwards for 1.5 s in a straight line in one of 8 directions (4 cardinal and 4 diagonal – 0, 45, 90, 135, 180, 225, 270, and 315 degrees) presented in random order. The stimulus sizes were chosen pragmatically to ensure clear visibility, stable fixation, and reliable tracking in a bedside, head-free clinical setting, rather than to target a narrow psychophysical regime. The relatively large target size (~1.5 dva) was chosen pragmatically to ensure clear visibility, stable fixation, and reliable tracking in a head-free bedside setting; this choice is consistent with prior pursuit studies using larger moving objects (23) work showing enhanced pursuit for larger stimuli (24), and recent paradigms using comparable similarly sized targets (25).

The trial sequence was repeated after a 0.5 s blank screen, with 2 trials per direction, and 16 trials per run of about 1 min. The target speed, when viewed from 60 cm, was ~4.4 deg./s in the horizontal and vertical directions and ~6.2 deg./s along the diagonals (with identical horizontal and vertical speeds). Note that all space measures in degrees (degrees of visual angle, dva) were set in screen pixels and converted here to dva based on the assumed 60 cm sitting distance, which slightly varied across individuals. The stimulus sequence, therefore, included predictable elements, such as fixed timing and constant-velocity linear motion, which were intentionally used to reduce task complexity and ensure feasibility in a bedside clinical setting. At the same time, the direction of motion was randomized across trials, requiring participants to initiate tracking toward an unpredictable trajectory on each trial. Thus, while the paradigm supports stable tracking once initiated, it retains an element of unpredictability at movement onset.

##### Experiment 2: smooth pursuit under occlusion

The paradigm was identical to that of experiment 1, except that the target was hidden by a bright ring of varying thickness (Figure 1b). There were two types of occluder tested in separate runs: (1) A visible occluder disc with luminance of 21.6 cd/m^2^, with 3 different thicknesses (2°, ~4°, and ~6°) in random order, one trial per direction, 24 trials for a run of ~1.5 min. The visible-occluder run was always presented before the virtual-occluder run; (2) A virtual occluder, which was the same as the visible occluder, except that it was black as the background and therefore was invisible; its border was uncovered only when the target moved under the occluder. During the occlusion phase, the predictable motion trajectory may support performance through short-term extrapolation, and occlusion-based measures should therefore be interpreted as reflecting both tracking maintenance and predictive processes.

#### Procedure

The nature of the study was explained to the participants, and all participants provided their informed consent to participate. The standard Tobii calibration was performed before each session. The participants were seated about 60 cm from the screen. The experiments were conducted with a free head to enable hospital testing under convenient conditions, including wheelchairs. Therefore, the actual sitting distance varied between observers and trials (see the Data Analysis and Results). The task was to track the moving target, without any other requirement. Each experimental run lasted for ~1 min for experiment 1 and 1.5 min for each part of experiment 2, with a total of ~5 min. The TBI participants performed the experiments 3 times, on average, on different days during their hospitalization, whereas the control participants did the experiments once. The clinical condition of the patients was assessed via the “Functional Independence Measure” (FIM) (26, 27), which is used in the LRH hospital as a clinical standard for functional evaluation. Functional status was assessed using the Functional Independence Measure (FIM), which is routinely administered as part of standard clinical care at Loewenstein Rehabilitation Hospital. For each eye-tracking session, the FIM score closest in time to the recording date (either before or after) was assigned. For participants who underwent multiple eye-tracking sessions over the rehabilitation period, analyses requiring a single functional value per participant were based on the average of the FIM scores corresponding to the days on which eye-tracking data were collected. Admission and discharge FIM scores were not averaged unless they coincided with an eye-tracking session. This approach was chosen to ensure that functional status reflected the patient’s clinical condition at the time of oculomotor assessment, rather than relying on admission or discharge scores that may not accurately represent interim functional fluctuations.

For comparison, the Glasgow Coma Scale (GCS) (28), a standard severity measure determined during patients’ admission, was also used. In addition, dominant hand data and the direction of the head impact during the injury were collected.

### Data analysis

Preprocessing: Because the experiments were conducted with a free head (see Procedure), the actual viewing distance varied between observers and trials. For each trial, we calculated the viewing distance as the average distance during the interval from −0.5 s to 0 s relative to the start of tracking, excluding outliers (>2 SD). This distance ranged from 40 to 95 cm; more than 75% of the trials fell between 50 and 70 cm. Trials outside the 40–80 cm range were discarded. On average, the TBI group sat 58 cm from the screen, compared with 65 cm in the control group. The closer proximity in the TBI group was largely due to some participants using wheelchairs, which required adjustments in sitting positions for accessibility and comfort. Since target motion was defined in screen pixels, we normalized the eye-tracking data by converting them to degrees of visual angle using a fixed sitting distance of 60 cm. This ensured that data could be meaningfully compared across participants.

Blink and artifact rejection: Blinks were detected via pupil size loss and verified by vertical eye position deviations (>4 SD), following the protocols established in our previous studies [e.g., (23)]. Periods of missing data, including blinks and tracking loss (e.g., head movements), averaged 21% across participants, with no significant differences between groups.

Saccade detection: Saccades were detected using a velocity-threshold procedure based on Engbert and Kliegl (29). Although originally developed for microsaccades, this adaptive framework is widely used to detect general saccadic events when parameters are appropriately adjusted. Given the moderate sampling rate (90 Hz) and spatial precision of the Tobii 4C, we applied velocity bounds of 8–400°/s to exclude noise while retaining tracking-related catch-up saccades. Only a small proportion of candidate events fell outside these bounds, primarily reflecting obvious artifacts (e.g., blinks or large reorienting movements). We did not impose the conventional lower peak-velocity cutoff (e.g., 30°/s) because moderate sampling rates can attenuate estimated peak velocities for small saccades. Consistent with this, 99.9% of detected events exhibited peak velocities above ~20°/s. Similarly, no lower amplitude threshold was imposed, as our trial-averaged summary measures are relatively insensitive to occasional false detections. To exclude large reorienting movements or pursuit resets, an upper amplitude threshold of 8° was applied. As empirical validation, detected events exhibited the expected main-sequence relationship, and the temporal profile of group differences remained stable across a broad range of minimum amplitude thresholds. Finally, tracking precision (RMS sample-to-sample noise) did not differ significantly between groups, ensuring comparable detection sensitivity (see Results).

Smooth and saccadic pursuit analysis: To quantify the contribution of catch-up saccades to target tracking (*here termed “saccadic pursuit”*), we derived a measure of catch-up saccade gain as follows. First, we removed the effect of saccades from the traces by erasing saccade periods (set to NaN in Matlab) and aligning points before and after each saccade by subtracting the saccade-induced displacement from the subsequent trace. We then subtracted the saccade-removed traces from the full traces to obtain the saccade-based component of tracking. Pursuit speed for both the smooth and saccade-based components was estimated using linear fits computed with the Matlab statistical toolbox function *robustfit*, with outlier rejection (weights <3 SD from the mean).

Additional oculomotor parameters: First, we computed a set of 6 oculomotor measures described in Table 3; the exact method of calculating each measure is explained in the table. All measures were calculated per epoch and then averaged across epochs per participant and condition; outliers that exceeded 2 Standard Deviations from the mean were removed, repeated twice. Group averages were calculated across participants without outlier rejection.

**Table 3:** Oculomotor measures of TBI.

Measure	Short description	Details
SacPurs (A)	Saccadic pursuit Gain	The saccadic (catch-up) pursuit is the saccadic component of the tracking that complements the smooth part. It is calculated per epoch as the speed (the slope of the linear fit in the 1–1.6 s post-stimulus onset time window) of the difference between two traces: full tracking minus the smooth tracking part (after saccades are removed and data aligned, Figure 2a), transformed to gain (fraction) by dividing the stimulus speed (4.4 deg./s for H and V, 6.2 for diagonals). Lower saccadic pursuit implies a smoother tracking.
OccDev (B)	Occluder-induced deviation from unoccluded pursuit	Deviation from unoccluded tracking during occlusion (Experiment 2). Each tracking trace was baseline-normalized relative to time 0 and converted to an unsigned trace, allowing opposite movement directions to be combined. A common unoccluded reference trajectory was computed from Experiment 1, separately for horizontal and vertical components. The primary measure was based on the horizontal component of deviation, including horizontal and diagonal trials. For each occluded epoch, the signed deviation from the reference was averaged over the 0.4–1 s time window and then averaged across epochs within observer after outlier rejection (>2 SD) (see Methods: Occluder-induced deviation analysis).
1st Speed (C)	Initial track speed	The time course of total eye velocity (drift, pursuit, and saccades) was first calculated per epoch (See Methods). The maxima were then calculated in the window 250–350 ms post-stimulus and averaged across epochs per participant.
sacRT (D)	Initial sac. Latency (catch-up)	A measure of the saccade response time, calculated per epoch as the latency of the first saccade in a time window of 250–800 ms relative to the onset of the pursuit target. It measures the speed of the open-loop process, in a condition in which the target direction is unknown in advance.
Pupil (E)	Pupil dilation, constriction	Change in pupil size calculated per epoch as the average in a 200–750 ms time window, expressed in percentage relative to the baseline pupil size (−0.5 to 0 s time window).
IOLag (F)	Vergence instability, via interocular lag	The horizontal interocular lag between the leading and the other eye time course was first calculated per epoch, and then normalized to time zero (the stimulus onset). Then, the maxima in the 1,250–2000 post-stimulus interval were calculated and averaged across epochs.

Six oculomotor measures that reflect the quality of tracking in the two experiments are listed together with a description of their exact computation method. The measures were computed per epoch and then averaged across epochs per participant and condition with outlier rejection (see the Methods section).

Occluder induced deviation analysis: To combine opposite movement directions without sign cancelation, each horizontal and vertical tracking trace was first baseline-normalized by subtracting gaze position at time 0 (stimulus onset), and then converted to an unsigned trace by taking the absolute value. A common unoccluded reference trajectory was computed from Experiment 1 as the control-group average of these normalized unsigned traces, separately for the horizontal and vertical components. For each occluded epoch, deviation from this reference was computed component-wise. The primary analysis focused on the horizontal component, which provided the most stable and informative estimate across directions. Trials with a horizontal motion component (horizontal and diagonal directions) were included, whereas purely vertical trials were excluded. The signed deviation (in degrees of visual angle) was obtained by subtracting the reference trace from the corresponding unsigned trace and averaged over the 0.4–1 s time window. These values were then averaged across epochs within each observer after outlier rejection (>2 SD), separately for each occluder width. Because the unsigned transformation and reference construction were applied uniformly across all conditions, this procedure preserves relative differences between groups, and the resulting scalar deviation measure is not expected to depend on the specific choice of reference trajectory, apart from minor numerical differences due to missing samples and outlier rejection.

Saccade rate and eye velocity modulation functions: A saccade rate modulation function was computed to assess event-related eye velocity modulation (30), following the approach used in our previous studies (22, 31). The validity of this approach has been demonstrated in previous studies. The calculation was based on only saccades meeting the temporal criteria described above, which were included and proceeded as follows: for each epoch, the rate function was determined by convolving a value of ~90/s (corresponding to the sampling rate) with a Gaussian window with a sigma of 50 ms centered on the saccade onset. These rate functions were then averaged across epochs within participants, separately for each condition. To normalize the data, the participants’ mean was subtracted from each rate function, and the total average across all conditions and participants was added. Finally, the mean and standard error were recalculated across participants to derive the final modulation function. Note that the normalization process affected only the error bars and not the mean across participants.

We also computed an eye-velocity modulation function to quantify movement speed over time, including smooth pursuit, drift, and saccadic components. For each epoch, eye velocity was estimated in sliding 150-ms windows advanced in 22-ms steps. Within each window, velocity was defined as the maximal two-dimensional displacement, computed from the horizontal and vertical position ranges as $\sqrt{\Delta H^{2}+\Delta V^{2}}$ , divided by the window duration. Because diagonal motion contains both horizontal and vertical components and therefore has a larger vector displacement than cardinal motion for the same component speed, diagonal velocities were divided by $\sqrt{2}\;$ before averaging across directions. This normalization expressed all velocities in component-speed units and allowed cardinal and diagonal trials to be combined.

### Statistics

**Figure 2:**
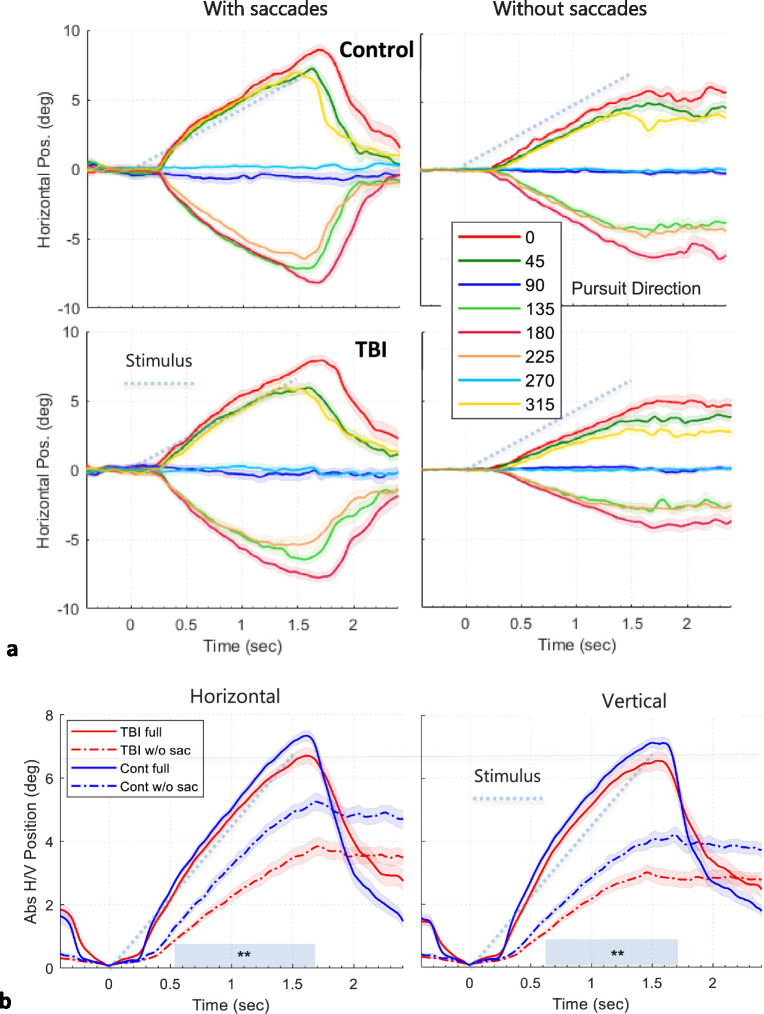
Results for smooth and catch-up saccade pursuit (experiment 1). **(a)** Horizontal gaze traces as a function of time, for each target direction, normalized by the pre-trial period, averaged across trials and then across observers, for the full traces (Left) and for traces without saccades (after saccade removal and alignment, Right), for the TBI and Control groups (in different rows). Error bars denote 1SE across observers. Note the much shallower traces, without saccades, in the TBI group. **(b)** Horizontal (left) and vertical (right) gaze traces, normalized and averaged across all relevant directions (shown in panel **(a)**) in absolute values, with (solid lines) and without saccades (dashed lines) for the two groups. The smooth (without saccades) part of tracking (the dashed lines) was compared between groups using a permutation test, yielding *p* < 0.001*** for the marked gray bar in both the horizontal and vertical traces. The light blue dotted lines show the stimulus position for the rightward and upward movement in panel **(a)**, and all movements in panel **(b)**.

**Figure 3:**
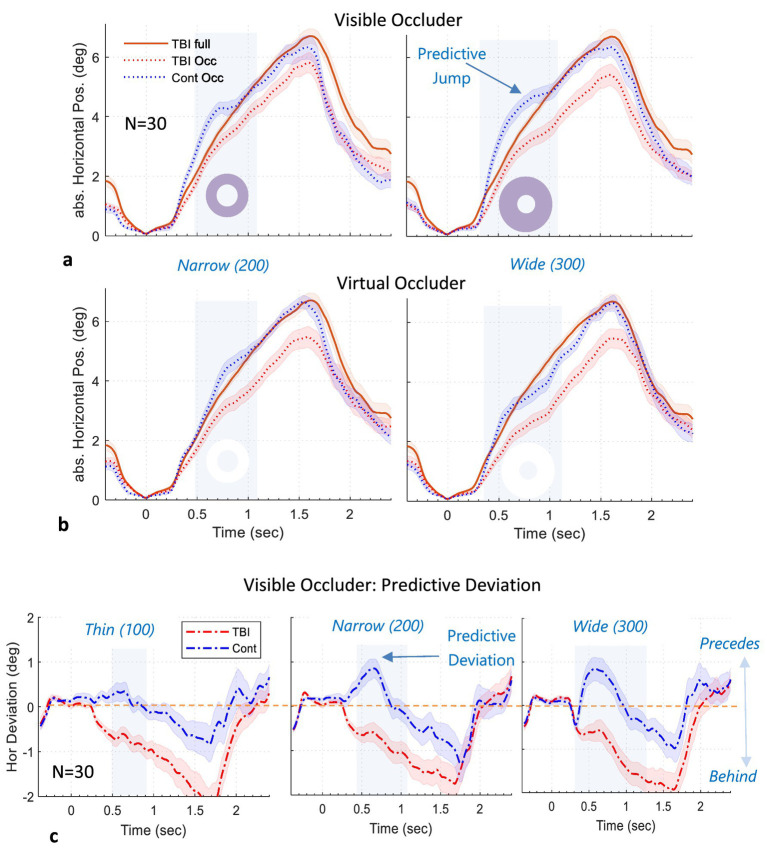
Results for smooth pursuit under occlusion (experiment 2). **(a,b)** Horizontal gaze trace averages (all directions except vertical) as a function of time, normalized by time 0, in absolute values, averaged across trials and then across observers (*n* = 30, 1SE across subjects’ error sleeves), for the TBI (red) and Control (blue) groups in dotted curves. The red solid lines plot the TBI group average of unoccluded tracking traces from Figure 2b for reference (the Control is similar and omitted for clarity). **(a)** Visible occluder, **(b)** Virtual occluder. **(c)** The occluder induced deviation from the unoccluded horizontal traces (all directions except vertical) for the TBI (red) and Control (blue) groups (corresponding to the difference between the occlusion traces in panel **(a)** and the unoccluded group reference, see the Methods section). Negative values correspond to lagging. Note the positive deviation of the Control group in panel **(a)** that precedes the target (especially for the wide occluder, denoted by an arrow, not found for the virtual occluder in panel **b**), whereas the TBI groups lag behind. This deviation effect is more explicitly shown in panel **(c)**.

**Figure 4:**
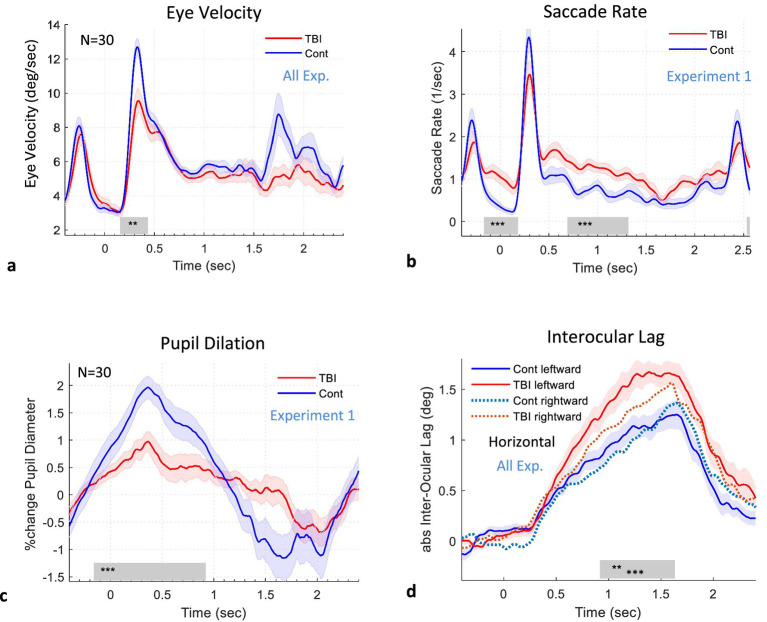
Additional tracking time-course properties, a group comparison. **(a)** Eye Velocity (including saccades, drift, and pursuit) time course averaged across all experiments. Note the faster velocity of controls around the time of the initial catch-up saccade. **(b)** Saccade rate modulation in Experiment 1. Note the lower pre-trial rate in the controls, reflecting anticipatory inhibition in preparation for the stimulus onset, which is reduced in the patients. **(c)** Pupil dilation in experiment 1, reflecting the transient recruitment of arousal for starting the tracking, expressed in percentage relative to the pupil size before target onset, averaged across participants (*n* = 30). **(d)** The horizontal interocular lag between the leading and other eyes (the gaze difference in absolute values reflecting increasing eye lead), normalized to time zero (stimulus onset) is plotted as a function of time for the horizontal tracking components, averaged across participants, separate for the leftward and rightward tracking. Only trials with a sitting distance of 50-70 cm were included. Note the higher interocular lag for the patient (red), even more for leftward tracking. In all plots, the gray bars denote the period of a significant difference (the nonparametric permutation test, see the Methods section) and the error sleeves show 1SE across participants.

Statistical analyses were performed using MATLAB version 2024b. Unless otherwise stated, significance was defined as *α* = 0.05 (two-sided). Statistical analyses were conducted primarily on participant-level summary measures, obtained by first averaging across valid epochs within session and then across sessions within participant, such that each participant contributed a single value to all between-group analyses. Most analyses were conducted on discrete oculomotor measures rather than on the raw time series, except for the waveform analyses shown in Figures 2b, 3, 4.

For waveform comparisons of tracking traces, pupil responses, interocular lag, and saccade-rate modulation, we used a cluster-based nonparametric permutation test (32) as applied in our previous study (33). At each time point, an independent-samples t-statistic was computed between the TBI and control groups, and contiguous suprathreshold time points were grouped into clusters. Cluster-level statistics were calculated as the sum of t-values within each cluster. Statistical significance was assessed by permuting group labels across participants 1,000 times, recomputing the maximal cluster statistic for each permutation, and comparing the observed cluster statistic with the resulting null distribution. The *p*-value was defined as the proportion of permutations in which the maximal permuted cluster statistic exceeded the observed value.

**Figure 5:**
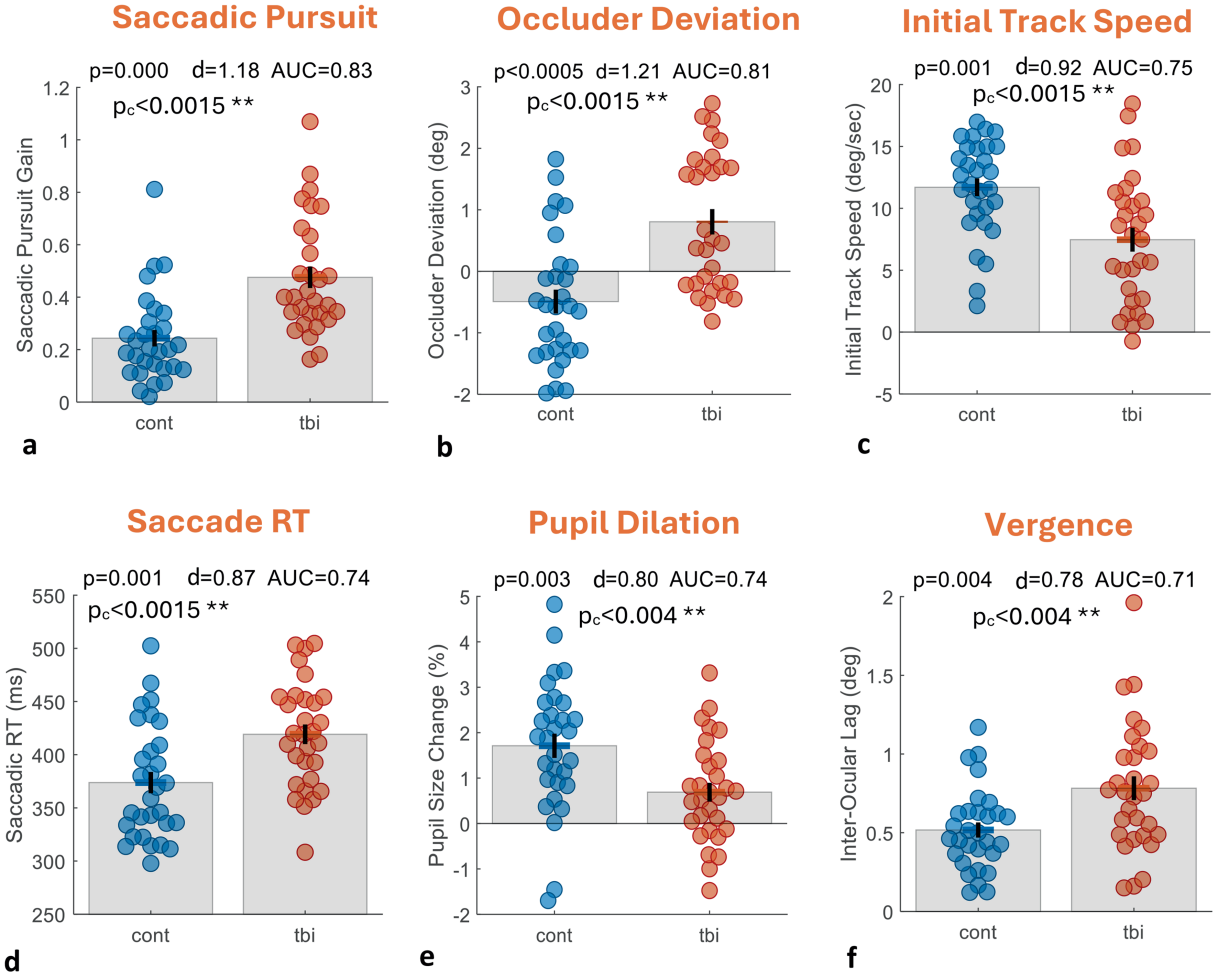
Oculomotor measures of TBI. Group differences for different oculomotor parameters (described in Table 3). **(a)** saccadic pursuit gain, **(b)** occluder deviation, **(c)** initial track speed, **(d)** saccade RT, **(e)** pupil dilation, and **(f)** vergence instability. For each parameter, a “bee-swarm” plot is shown with a dot per participant (*N* = 30 per group). The error bars denote 1SE and the discrimination results are marked for each plot: p = *p*-value (t-test, uncorrected), pc = p-value FDR-corrected, d = effect size (Cohen’s *d*), AUC = the area under the ROC curve. As shown, all parameters showed a significant difference between groups (even when corrected for multiple (6) comparisons).

**Figure 6:**
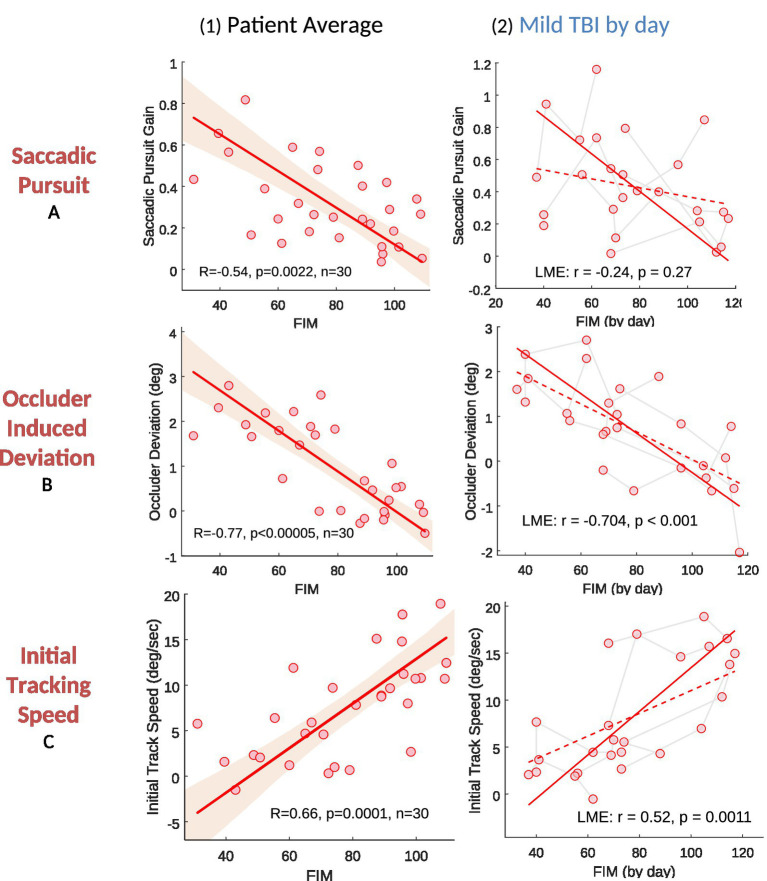
Oculomotor measures in relation to patients’ functional status. Three oculomotor measures are shown in separate rows (see Table 3): **(A)** saccadic pursuit gain, **(B)** occluder-induced deviation, and **(C)** initial tracking speed (initial “catch-up”). For each measure, its association with the Functional Independence Measure (FIM) is presented in two columns: (1, left) patient averages across days (one marker per patient), and (2, right) repeated measurements in the mild TBI subgroup, in which each patient (*n* = 7) was assessed 3–4 times (total n ≈ 25). In the right column, markers from the same patient are connected by light-grey lines. For the left column, Pearson correlation coefficients (R) and *p*-values from orthogonal regression (see Methods) are shown without outlier exclusion. For the right column, Linear Mixed-Effects (LME) analysis was used to account for repeated measurements within patients; both the orthogonal regression line (solid) and the LME regression line (dashed) are shown. All correlations were significant except for panel **(A-2)**. In panels **(B-2,C-2)**, within-patient trajectories generally followed the overall direction of association, consistent with changes observed during rehabilitation. However, these repeated-measures results are limited to a small subgroup and should be interpreted as exploratory. Accordingly, the figure supports an association between oculomotor performance and functional status, but does not by itself establish longitudinal monitoring utility or prognostic value.

**Figure 7:**
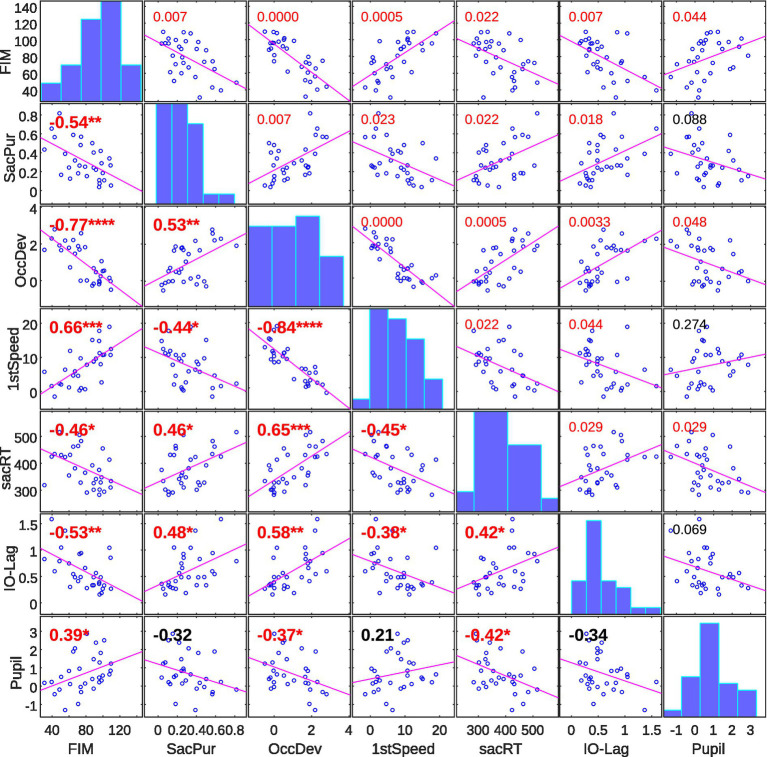
Correlation matrix of the oculomotor measures of smooth pursuit. Six oculomotor indices are correlated with each other, as well as the patient’s condition (FIM average _enter_, _leave_). Each dot represents one patient. The Pearson correlation measures (R) are shown in the lower part of the correlation table, whereas the p-values (FDR-corrected for multiple comparisons, see the Methods section) are shown in the upper part. Significant correlation values are denoted in red with the standard star notation. The correlations between the patient’s condition and the oculomotor measures are shown in the leftmost column, showing that with higher (better) FIM, the TBI patients showed (1) less non-smooth “saccadic pursuit”, (2) smaller occluder-induced deviation from un-occluded tracking, (3) a shorter latency of the 1^st^ catch-up saccade, (4) less vergence instability (interocular lag), and (5) larger initial pupil dilation. All measures were significantly correlated with the clinical assessment (FIM, R=~0.7, p<0.0005, FDR-corrected) and between each other. Age was not correlated with the patient’s condition but was modestly (significantly) correlated with the saccade RT and the occluder-induced deviation.

For correlation analyses at the participant level (Figures 6, 7), Pearson’s correlation coefficient (R) was computed without excluding data points. Orthogonal regression was used for visualization to account for measurement error in both variables. Multiple comparisons in Figures 5–7 were controlled using the false discovery rate (FDR) procedure (34). For group-level discrimination analyses (Figure 5), receiver operating characteristic (ROC) analysis was used to compute the area under the curve (AUC). Group differences were assessed using independent-samples t-tests, and effect sizes were reported as Cohen’s d. Confidence bands around regression lines were estimated using nonparametric bootstrap resampling (1,000 iterations). For each bootstrap sample, an orthogonal regression line was fit, and the 95% confidence band was defined by the 2.5th and 97.5th percentiles of predicted values across resamples.

## Results

We computed 6 oculomotor measures that we analyzed for group differences and the relation to the severity of TBI. Although there were 2 experiments, we first presented the results by oculomotor measure, with some measures accumulated across experiments (Figures 2, 4, 6, 7). Then we analyzed the inter-relationships between measures and the cross-measure effects (Figures 6, 7).

Eye-tracking quality and basic measures. To ensure data quality was comparable between controls and TBI patients despite the use of a remote eye tracker, we first analyzed tracking reliability and basic oculomotor characteristics.

Data yield and precision: The percentage of valid gaze samples (excluding blinks and off-screen artifacts) was high across the cohort (M = 80%, SD = 15) and did not differ significantly between groups (*t (60) = 1.34, p = 0.34*). Spatial precision, quantified as the Root Mean Square (RMS) of sample-to-sample (S2S) deviations during fixation, was calculated to assess instrumental noise. Median RMS sample-to-sample gaze noise was 0.09° (IQR: 0.05°) in the TBI group and 0.10° (IQR: 0.06°) in controls, with no significant difference between groups (Wilcoxon rank-sum test, *p* = 0.06; two-sample t-test on subject-level means, *p* = 0.4). This confirms that group differences in pursuit dynamics were not driven by tracking artifacts or signal noise differences.

Basic oculomotor measures: We also verified that the groups were matched on fundamental oculomotor traits. There was no significant difference in spontaneous Blink Rate (*M_TBI_ = 12.6/min vs. M_cont_ = 10.8/min; p = 0.5*). Similarly, the average amplitude of saccades during the pursuit task was comparable between groups (M*_TBI_* = 1.12^o^ vs. *M_cont_ =* 1.15 ^o^ and p = 0.5), indicating that both groups engaged similar global scanning strategies despite the expected differences in pursuit dynamics.

### Saccadic pursuit (catch-up saccade contribution)

The results of the basic smooth pursuit task (experiment 1) are shown in Figure 2. First, we plotted the group average horizontal traces for different directions (Figure 2a) for the full tracking (left column) and without catch-up saccades (right column), where saccades were removed and data aligned (see the Methods section). As shown (Figure 2a, left panel), both groups tracked the target similarly with a gain that approaches 1 (compared to the stimulus time course in a dotted line). To quantify this similarity, we computed the gain for each participant, averaged across trials of all directions with outlier trials rejected (outside the 0.3–3 gain range). We found an average gain of 0.88 (SD 0.14) and 0.92 (SD 0.13) for the control and TBI groups, respectively, with an insignificant difference between groups.

The results of the smooth part of tracking with traces after removing the catch-up saccades are shown in Figure 2a, right column. As shown, both groups used catch-up saccades, consequently making the smooth part of tracking (right column) appear “slower” in speed. However, unlike the full tracking, the smooth part of tracking was smaller in the TBI patients, as shown in Figure 2b (averages across directions in absolute values) for both the Horizontal and Vertical traces, comparing the solid lines (full tracking) and the dotted lines (smooth part, w/o saccades) between groups. We conducted a nonparametric permutation test (see the Methods section) to compare traces in the tracking time interval and found no difference between groups for the full tracking, and a highly significant difference for the smooth part of the tracking (*p* < 0.001*** for both horizontal and vertical traces). We termed the saccadic part of the tracking “saccadic pursuit” and estimated its gain, as described in Table 3 (item A). The results for the group comparison are shown in Figure 5a, showing a higher saccadic pursuit gain (~0.5) for the patients compared with the controls; there was a highly significant group difference (*p* < 0.0015 after FDR correction, an Effect Size of 1.18, and an AUC of 0.83).

### Occluder-induced deviation

An occluder, added in experiment 2, altered the horizontal and vertical traces compared with the unoccluded traces in experiment 1, causing a deviation that we assessed to determine a possible difference between groups. Three sizes of an occluding ring were tested in random order, in two types of the occluder, visible and virtual, tested in separate runs (see the Methods section). The main results of these experiments are presented in Figure 3, showing the horizontal position time course for the different directions for the two groups averaged across all non-vertical directions in absolute values, for the visible (3a) and virtual (3b) occluders. For reference, we also plotted the TBI group average of an unoccluded tracking trace from Figure 2b (the control is similar). As shown in Figure 3a, the control group “jumps ahead” when reaching the occluder, presumably a “predictive jump’, or a fast movement forward to wait for the reappearance of the target behind the occluder, upon which the tracking resumes in the typical way. The “jump ahead” effect is strongly attenuated in the Virtual condition, in which the exit point is less clear (Figure 3b). In comparison, the TBI group shows no “jump ahead” effect and resumes tracking after the occlusion, lagging, with no apparent difference between the Visible and Virtual occluders.

The difference between groups is more explicitly demonstrated in Figure 3c by plotting the occluder-induced deviation from the unoccluded horizontal traces (all directions except vertical), corresponding to the difference between the occlusion traces in Figure 3a and the unoccluded group reference (see the Methods section). Note the negative values for the TBI group, which implies lagging, and the positive deviation of the Control group that precedes the target.

To assess the occlusion effect at the group and individual level statistically, we extracted a scalar measure of “Occluder-induced Deviation” (OccDev), described in Table 3 (item B). The results for the group comparison, based on both the visible and invisible occluder data, are shown in Figure 5b, showing a higher occluder-induced deviation from unoccluded tracking (averaged in 0.4–1 s post-stimulus) for the patients compared to the controls; there was a highly significant group difference (*p* < 0.0015 after FDR correction, an Effect Size of 1.32, and an AUC of 0.84).

### Initial tracking speed

We derived a discrete measure quantifying the initial tracking speed. First, we computed the time course of the eye velocity, including saccades, drift, and pursuit (see the Methods section). The results, shown in Figure 4a, are pooled from all data of both the smooth tracking and occlusion experiments and from all directions. As shown, following an inhibitory period of about 150 ms post-stimulus, an initial catch-up movement started around ~200 ms for both groups, peaking around ~330 ms, with a higher peak for the controls. Then, the eye velocity decreases to the tracking speed (the average of cardinal and diagonal directions, also affected by the occluder in experiment 2); then there is another higher peak around ~1750 ms, jumping back to fixation, even more in the controls. We conducted a nonparametric permutation test (see the Methods section) that showed a significant difference around the first peak (~200–400 ms).

To assess the initial eye velocity (shown in Figure 4a peak) at the group and individual levels statistically, we extracted a scalar measure of the “Initial Track Speed” (the 1st Speed) as described in Table 3 (item C). The results for the group comparison are shown in Figure 5c, revealing a higher initial movement (catch-up) speed for the controls compared with the patients, along with a highly significant group difference (*p* < 0.0015 after FDR correction, an Effect Size of 0.92 and an AUC of 0.75).

### Saccade RT and inhibition

First, we analyzed the saccade rate modulation (see the Methods section); the results are shown in Figure 4b. As shown, the control group inhibited the saccades before the onset of the target, reflecting temporal anticipation, which was much weaker in the patients. To assess the significance of this effect, we conducted a nonparametric permutation test (see the Methods section), which revealed a significant difference (***) in the pre-stimulus period and immediately after (~ − 200–200 ms, Figure 4b). There was also a highly significant difference during the tracking period, consistent with the “saccadic pursuit” measured, described above. Importantly, the observed temporal profiles and group differences were stable across a wide range of saccade amplitude thresholds (0.05–0.6°).

We conducted an additional analysis of the latency of the initial catch-up saccade, which we termed “Saccade RT,” described in Table 3 (item D). Since in all experiments the target appeared at fixation and started moving in one of 8 directions unknown to the observer, the measure of Saccade RT represents the processing speed of identifying the direction of the target and starting the tracking (the open-loop stage). The group comparison for data from all experiments is shown in Figure 5d, showing an earlier initial saccade for the controls compared to the patients (374 *vs.* 420 ms), with a highly significant group difference (p < 0.0015 after FDR correction, an Effect Size of 0.87, and an AUC of 0.74). Note that although in the saccade rate modulation function (Figure 4b) there is no apparent difference in the initial peak (around 300 ms), the saccade RT is based on the latencies of the initial saccades in an interval of 250–850 ms, and many saccades were delayed in the patients. Although small-amplitude saccades are measured with higher relative noise at 90 Hz, Saccade RT is dominated by the latency of the first directionally appropriate catch-up saccade following target motion onset, making this measure robust to occasional early or non-directional small saccades.

### Pupil dilation

The initial catch-up saccade was found to be associated with dilation of the pupil, which reflects recruitment of arousal to drive the overt attention shift (35). First, we analyzed the pupil size modulation time course for the two groups; the results are shown in Figure 4c. As shown, the pupil dilated relative to the pre-stimulus baseline, peaking around 350 ms post-stimulus, with ~2% dilation for the controls and only ~1% for the patients, an effect found highly significant (nonparametric permutation test, see the Methods section). Next, we extracted a scalar measure of the Pupil Dilation, as described in Table 3 (item E). The results for the group comparison are shown in Figure 5e, showing a higher pupil dilation for the controls compared with the patients, with a highly significant group difference (*p* < 0.004 after FDR correction, an Effect Size of 0.8, and an AUC of 0.74).

### Interocular lag

We observed that when the eyes start to track a target moving from fixation horizontally, they start deviating, compared with the pre-tracking time, with increasing interocular lag over time up to 1–2 deg (Figure 4d). We quantified this effect by subtracting the horizontal gaze position of the two eyes to compute the interocular lag, normalizing it to the interocular difference at time zero, and taking the absolute value to avoid the effect that the eye leads. In this way, we focused only on the deviation induced by the tracking. Note that for this analysis, we considered the individual sitting distance when converting the traces to degrees of visual angle (dva), although the results persist without this correction. The results are shown in Figures 4d, 5f, with data from experiment 1 (simple tracking). As shown, patients exhibited a larger interocular lag than controls in the horizontal component of tracking, and this lag increased during tracking. We quantified it via the discrete Interocular Lag measure, Table 3 (item F); group averages are compared in Figure 5f, showing a significantly larger interocular lag in the TBI group (p < 0.004 after FDR correction, an Effect size of 0.78, and an AUC of 0.71). There was also an interocular lag on the vertical movement, but this effect was much smaller and did not reach group significance.

### Comparing the oculomotor measures and the clinical condition

To assess the relationship between the oculomotor measures and the clinical assessment of the patients, we first considered the two clinically available measures, (1) the GCS, which was determined at the time of admission and does not reflect the current condition of the patient, and (2) the FIM (Functional Independence Measure, see the Methods section), which could change over time and was assigned per patient and on the day of testing.

First, we examined three measures in more detail: (1) the Saccadic Pursuit, (2) the Occluder-Induced Deviation, and (3) the Initial Tracking Speed. Their associations with the FIM are shown in Figure 6 using two complementary analyses: (a) participant averages across days (one value per participant), and (b) repeated measurements in the mild TBI subgroup (*n* = 7), with 3–4 assessments per participant. As shown, all correlations were significant except for saccadic pursuit gain in the repeated-measures mild subgroup analysis, and most remained significant after FDR correction (see Methods). Overall, these findings support an association between these oculomotor measures and current functional status. However, the repeated-measures analysis in the mild subgroup is limited and should be interpreted as exploratory.

To obtain a more complete picture of the relationships between the oculomotor measures and the patients’ condition, we computed a correlation matrix for the patient data that reveals the inter-relationship and consistency between the measures, as well as the clinical assessment and the age of the patient. The results are shown in Figure 7. As shown, the different oculomotor measures were remarkably correlated with the average FIM score computed across the eye-tracking assessment days for each participant, with absolute R in the range ~0.6–0.8, except for the pupil (R = 0.41) and *p*-values (after FDR correction) between 0.0001 and 0.045. The highest correlation was found for the Occluder-Induced Deviation (R = 0.78). In all measures, the direction of correlation was consistent with the group average comparison to controls (Figure 5), i.e., higher (better) FIM was associated with oculomotor measures that were similar to the healthy controls. All measures were mostly significantly correlated with each other, but with a lower correlation, indicating that these measures were not redundant.

The sitting distance, computed per trial at the start of tracking (see the Methods section), varied both across participants and within participants (due to free head movement). We excluded two control participants because they sat too far from the screen (an average distance >85 cm). To assess whether the sitting distance influenced our findings, we computed correlations between each participant’s average sitting distance and the parameters analyzed in Figure 5. There were no significant correlations, nor any indication of a trend, with p-values ranging from 0.2 to 0.9 in the TBI group and 0.5 to 0.7 in the Control group. Similarly, we found no significant correlation, nor any trend between the sitting distance and the individual average Functional Independence Measure (FIM) scores. These findings confirm that the observed differences between the TBI and control groups-and among patients themselves-cannot be attributed to variations in the sitting distance, thereby supporting the validity of our results. We also examined the correlation of the same measures with the GCS and found no significant correlation or trend for any of them (*p* > 0.5 for 4 of the measures, *p* > 0.15 for the pupil and msRT).

### Medication (Keppra) effects

Approximately half of the TBI patients were treated with levetiracetam (Keppra) during rehabilitation. To evaluate potential medication-related confounds, we compared patients treated with Keppra to those not treated on clinical measures (average FIM, GCS), eye-tracking quality, and the six oculomotor indices shown in Figure 5. No statistically significant differences were observed between the medication groups. For the six oculomotor measures (Figures 5a–f), *p*-values ranged from 0.07 to 0.52 (Cohen’s d = 0.09–0.70). Specifically, saccadic pursuit (*p* = 0.15, d = 0.54), occluder-induced deviation (*p* = 0.52, d = 0.24), initial tracking speed (*p* = 0.23, d = 0.45), saccade RT (*p* = 0.31, d = 0.38), pupil dilation (*p* = 0.18, d = 0.09), and vergence instability (*p* = 0.07, d = 0.70) did not differ significantly between medicated and non-medicated patients. The nominal trend observed for vergence instability did not reach statistical significance and should be interpreted cautiously. Similarly, no differences were found for eye-tracking quality measures (*p* = 0.48, d = 0.26), average FIM (*p* = 0.67, d = 0.16), or GCS (*p* = 0.48, d = 0.27). These findings suggest that the observed oculomotor differences are unlikely to be explained by Keppra treatment in this cohort.

## Discussion

Oculomotor behavior is generally known as a good indicator of the severity and condition of TBI patients (36). In our study, we used a simple smooth pursuit paradigm using remote bedside eye-tracking technology that was found to produce sensitive markers of oculomotor dysfunction associated with traumatic brain injury. In the results of the first experiment with the standard linear pursuit movement, we found a difference between the TBI and the control groups in several measures of the pursuit quality. In the results of the second experiment with the pursuit movement under a visible occluder and an occluder assimilated into the background, the patients exhibited a larger deviance induced by the occluder.

### The oculomotor indices of TBI

We developed 6 oculomotor measures or indices to quantify the quality of eye movements during linear pursuit as a marker for TBI. These measures included the saccadic pursuit, tracking deviation under occlusion, initial tracking speed, initial saccade latency, pupil response, and vergence instability. The analysis of the indices revealed significant differences between the healthy and TBI groups (Figure 5). The FIM index was correlated with all the oculomotor indices with significance (R = ~0.4–0.8).

Beyond the observed deficits in smooth pursuit, our findings highlight a significant increase in interocular lag in TBI patients, particularly during horizontal tracking (Figures 4d, 5f). This suggests a disruption in binocular coordination, potentially reflecting an impairment in the vergence mechanisms or in the asymmetric neural control of both eyes. Similar disruptions in interocular coordination have been observed in macular degeneration (MD) patients, where smooth pursuit deficits and altered binocular coordination can emerge due to reliance on peripheral vision and impaired binocular integration (37, 38) These parallels suggest that interocular lag may serve as a general marker of impaired sensorimotor integration across different neurological and visual conditions. Such disruptions may contribute to visual instability and difficulty in tracking moving objects in daily life, particularly in dynamic environments where precise binocular coordination is required.

Our findings indicate abnormal tracking behavior during occlusion in TBI patients. Unlike healthy controls, these patients failed to show the predictive ‘jump ahead’ eye movement that typically anticipates where and when the target will reappear after passing behind an occluder (Figure 3). This pattern is consistent with a disruption in motion-based prediction mechanisms, which in healthy observers can guide behavior even when motion information is partially unavailable or interrupted (39). In addition, anticipatory tracking depends on temporal expectations about *when* the target will reappear, and the pre-stimulus oculomotor inhibition has been proposed as an objective marker of temporal expectation (9, 10). A previous study by (12) used a virtual occluder (gap) with circular smooth pursuit and found increased tracking errors in chronic mild TBI patients, specifically during target occlusion, but not during unoccluded tracking. Interestingly, they observed via MEG increased beta amplitude in the control group across several parietal regions, presumably reflecting the increased cognitive effort or attention required to make a predictive jump. Such cognitive effort should theoretically be reflected in increased pupil dilation. In our study, we found increased pupil dilation in the control group during simple tracking (Figure 4c). However, the bright occluders in our paradigm induced a strong pupil constriction response that complicates the interpretation of these findings.

Our positional deviation analysis reveals a stark divergence in anticipatory control between cohorts during visual occlusion (Figure 3). While healthy controls successfully utilized internal predictive mechanisms to generate an anticipatory spatial lead to compensate for the occluded trajectory (40, 41), the TBI cohort demonstrated a pronounced negative deviation, progressively falling behind the target. This anticipatory shift in healthy tracking is consistent with the generation of predictive saccades during target blanking, a process where ongoing smooth pursuit enhances subsequent motion prediction (11, 42). Conversely, the failure to maintain spatial synchrony in the TBI group aligns with findings that traumatic brain injury impairs the extraretinal mechanisms required to maintain an internal model of motion during visual gaps. The absence of this anticipatory lead suggests not merely a failure to integrate velocity information, but a breakdown in the internal forward model-a predictive process that allows the brain to estimate future sensory states based on motor commands and previous experiences (43, 44). Ultimately, this supports the notion that TBI, often involving diffuse axonal injury, severely affects the higher-order cognitive control networks responsible for predictive inference, forcing patients to rely on reactive rather than anticipatory tracking strategies.

Because antiepileptic medication may influence oculomotor and pupillary dynamics, we examined whether levetiracetam (Keppra) treatment was associated with the observed effects. No significant differences were found between medicated and non-medicated patients for clinical measures or any of the six oculomotor indices. A nominal trend was observed for vergence instability, but this did not reach statistical significance and should be interpreted cautiously. These findings suggest that the reported oculomotor abnormalities are unlikely to be primarily driven by Keppra treatment in this cohort.

### GCS versus functional measures in TBI assessment

Although the Glasgow Coma Scale (GCS) is widely used to classify TBI severity, particularly in defining mild TBI (e.g., (12)), it primarily reflects the level of consciousness at the time of injury and may be less informative with respect to functional status during later stages of rehabilitation. In the present study, no significant correlation was observed between initial GCS scores and functional independence as measured by the FIM (*p* = 0.8), despite strong associations between FIM and multiple oculomotor measures (Figure 7). Consistently, none of the six oculomotor indices showed a significant correlation with GCS (mean *p* = 0.59, range 0.15–0.99; see Results). These findings suggest that, in this rehabilitation-stage sample, functional and task-based measures may better reflect patients’ current clinical status than acute injury severity indices alone.

### Comparison to previous studies

Although previous research has extensively studied eye movements in TBI, most studies have focused on mild TBI cases classified by GCS scores rather than functional status (13–15, 45–48). Our study demonstrates that simpler experimental methods can provide clinically relevant measures. Previous studies used more complex approaches such as circular tracking (12–15, 45, 46), target under occlusion (12), or sustained sinusoidal tracking (13). In contrast, we found that a simple repeated linear tracking task with 0.5-s pauses between segments was sufficient to reveal significant group differences. This simplified approach reduces patient fatigue and data complexity, making it more suitable for clinical applications. Additionally, our use of portable eye tracking without head restraints enabled comfortable bedside testing in rehabilitation settings, a crucial factor for clinical adoption. Finally, whereas previous studies mainly identified group differences between TBI patients and controls without establishing links to functional outcomes, our study demonstrates strong correlations between eye-tracking measures and FIM scores, directly connecting oculomotor dysfunction to functional independence.

### Limitations

Several limitations should be considered. First, the remote 90 Hz eye tracker limits single-trial temporal precision, particularly for latency-based measures and binocular timing estimates. While these measures become robust at the subject level through trial averaging, they should be interpreted as clinically oriented markers rather than high-precision physiological timing metrics. Second, the Tobii 4C was used in a head-free bedside configuration, and viewing distance varied across and within participants, with a group difference in average viewing distance. Although this variability was a deliberate tradeoff to preserve clinical feasibility, analyses reported in the Results indicated that viewing distance did not account for the observed group effects or correlations with the clinical measures. Third, controls were tested once whereas patients were tested across multiple sessions; although between-group analyses were conducted on participant-level averages, practice or familiarity effects cannot be fully excluded. Fourth, the visible-occluder run always preceded the virtual-occluder run, so order-related effects between occluder conditions cannot be fully excluded. Fifth, the groups differed in sex composition, with a predominantly male TBI cohort and a sex-balanced control group. This distribution reflects the epidemiology of TBI, but it also means that potential sex-related effects cannot be excluded and should be examined more systematically in future studies with larger and more balanced samples. Finally, although some patients were tested repeatedly during rehabilitation, the present study was not designed as a formal longitudinal or prognostic study, and most of the reported evidence is based on between-group differences and associations with existing functional status.

### Potential for future diagnosis and prognosis of TBI

Our results suggest that a simple and short bedside recording of a linear visual tracking examination of a few minutes could serve as an effective tool for providing measures of inefficient brain functioning after traumatic brain injury. Our detailed assessment of short and effortless visual tracking revealed markers that are very sensitive to the patient’s condition and therefore can be useful for clinical evaluation. These markers can be used in the future to reveal injury-specific deficits. For example, a horizontal interocular deviation could indicate an injury to the 6th nerve in the eye opposite the affected hemisphere. Moreover, these measures, combined perhaps with machine learning techniques, could be used to develop a good prognosis and evaluation of the efficacy of treatments. Future research should focus on combining reliable measures to develop practical, fast, and affordable user-friendly tools for doctors and professionals.

Beyond TBI assessment, our findings highlight the diverse nature of oculomotor impairments following traumatic head injury, which affect the initial tracking speed and latency, vergence instability, pupil-linked arousal, as well as the ability to predict and adjust to dynamic visual stimuli. These deficits may contribute to everyday challenges, such as reading or navigating dynamic environments. Our results suggest that fundamental eye movement behaviors- such as smooth pursuit and saccadic adjustments- could serve as useful indicators of these impairments, underscoring the potential value of incorporating eye-tracking into clinical evaluations. These findings support the potential of brief bedside eye-tracking measures as objective markers of current functional status during rehabilitation. Future longitudinal studies will be needed to determine their value for prognosis, monitoring change over time, and treatment-response assessment.

## Data Availability

The datasets generated and analyzed for this study are available from the corresponding author on reasonable request.
